# The role and implications of mammalian cellular circadian entrainment

**DOI:** 10.1002/1873-3468.70223

**Published:** 2025-11-20

**Authors:** Priya Crosby

**Affiliations:** ^1^ Institute of Cell Biology, School of Biological Sciences University of Edinburgh UK

**Keywords:** cell biology, cell signalling, circadian, entrainment, PERIOD

## Abstract

The ability to align circadian phase to specific cues, or ‘entrainment’, is a defining feature of a circadian rhythm. Entrainment is critical for useful circadian function, as it enables organisms to determine the specific time of day to perform temporally restricted behavioural and physiological activities, ranging from sleep to cell division. While mammals have long been known to entrain their circadian rhythm, recent work has shed light on how this is achieved in every single cell, all of which maintain their own individual circadian oscillation. Here I will highlight the current understanding of how the major entraining cues of light, feeding and temperature are communicated to cells to alter their phase. Knowledge of the mechanisms of cellular entrainment has the capacity to impact both fundamental understanding of circadian rhythms and our application of cellular circadian research to real‐world problems, including shift work.

## Graphical abstract

At their most fundamental level, mammalian circadian rhythms occur inside every individual cell. To tell the correct time, cells must align (or ‘entrain’) their circadian rhythm to the external environment. In this review, we highlight how cells entrain to the major circadian cues of light, feeding and temperature, and the implications this has for our understanding of circadian biology.

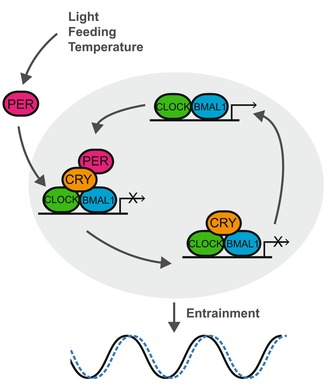

## Abbreviations

**BMAL1**, basic helix–loop–helix ARNT‐like protein 1

**CIRP**, cold‐inducible RNA‐binding protein

**CLOCK**, circadian locomotor output cycles protein kaput

**CREB**, cyclic AMP response element‐binding protein

**GCGR**, glucagon receptor

**GRE**, glucocorticoid response element

**HPA axis**, hypothalamic–pituitary adrenal axis

**HSF1**, heat shock factor 1

**mTOR**, mammalian target of rapamycin

**m‐uORF**, minimal upstream open reading frame

**PER**, PERIOD protein

**PI3K**, phosphatidylinositol 3‐kinase

**RBM3**, RNA‐binding motif protein 3

**SCN**, suprachiasmatic nucleus

**TGF‐β**, transforming growth factor beta

**TTFL**, transcription‐translation feedback loop

We live on a rotating planet. As a result, organisms across kingdoms have evolved their own internal biological ‘clock’, or circadian rhythm, to allow them to anticipate daily external changes. While we have known for over 50 years that some single‐celled organisms, notably cyanobacteria and the dinoflagellate *Gonyaulax polyedra*, exhibit competent circadian rhythms [1, 2], the prevailing hypothesis until the early 2000s was that circadian rhythms in mammals were primarily controlled by the suprachiasmatic nucleus (SCN), a specialised region of the hypothalamus that receives direct light information from the retina [3]. Over the last 20 years, a series of discoveries have challenged this view, and increasingly centre the individual mammalian cell as a critical regulator of circadian rhythmicity at higher physiological and behavioural levels.

This review will highlight the major discoveries that led to the identification of ubiquitous cellular circadian oscillators in mammals. I will set this within the current understanding of how the three major circadian entraining cues of light, feeding and temperature are communicated to the circadian oscillator inside every cell. Finally, I will discuss the importance and implications of cellular circadian entrainment for our understanding of circadian rhythmicity and circadian disruption.

## The identification of cellular circadian rhythms

The discovery of the suprachiasmatic nucleus (SCN) as a centralised neuroanatomical locus within the hypothalamus that detects rhythmic light cues and subsequently controls mammalian circadian behaviour was a major step for the circadian field. It provided, for the first time, a clear physiological basis for the well‐established phenomenon of circadian rhythmicity, which occurs at almost every level of mammalian biology [3, 4]. A critical offshoot of this discovery was that the identification of this highly rhythmic brain region allowed for targeted investigations of gene expression in this tissue. This work ultimately identified many of the core genes [5, 6, 7, 8, 9, 10, 11, 12, 13, 14] (*period*, *cryptochrome*, *clock*, *bmal1*)—sometimes termed ‘clock genes’—that are required for the maintenance of the circadian transcription‐translation feedback loop (TTFL) [15].

Following the discovery of these clock genes, it became increasingly apparent that their expression was not only restricted to the SCN, but that their corresponding transcripts could be found in tissues throughout the body [16]. Strikingly, several studies showed that rhythmic expression of clock genes and proteins persisted in organotypic slices of a wide range of tissues, including lung, liver and skeletal muscle [17, 18, 19]. These findings were, in many ways, analogous to the previous findings with organotypic slices of the SCN, which also show robust rhythms in expression of core circadian transcripts [11, 18]. A notable difference between these two systems is the vastly greater longevity of organotypic SCN slices, which can remain alive and correspondingly rhythmic in culture for many months [20]. In contrast, organotypic slices of other tissues do not survive so well in culture and show corresponding loss of rhythms over a period of days to weeks depending on culture conditions [18]. This ease of *ex vivo* culture has likely played no small part in the considerable success of the SCN as a model for studying various aspects of circadian control.

These early experiments using organotypic slices gave rise to an idea that mammalian circadian rhythms might be controlled by a series of ‘tissue clocks’ [17, 18, 21]. While this term is frequently not defined, many authors use it to refer to some kind of connection of the cells within specific organs or tissues, whose network forms a cohesive oscillator. This would be analogous to the SCN which, in addition to expressing the components of the circadian (TTFL), also has inter‐neuronal connections which in and of themselves produce circadian oscillations across the SCN [22]. This provides the SCN with an extra layer of circadian robustness, where cells within the SCN can communicate circadian timing information to their neighbours through both neuronal and paracrine signals [23]. It is worth noting that such strong communication of timing information between cells in a single tissue has not been demonstrated outside of the SCN.

Almost simultaneous with the discovery of tissue‐level oscillations in circadian gene expression, several studies described rhythms in cell lines grown in culture, with these findings initially described in Rat1 fibroblasts [16, 24, 25, 26], followed by additional single cell imaging in NIH3T3 cells [27]. Critically, these cellular rhythms were most readily observed at the population level when their phase was aligned through the application of cellular synchronising cues, such as serum shock or the synthetic glucocorticoid, dexamethasone [16, 24]. Inherently, this demonstrated that rhythms in cells outside the SCN conform to one of the three essential requirements of a true circadian rhythm [28, 29] in being entrainable to external cues. It also highlighted a critical difference between rhythms in cells and those that had been previously studied in the SCN: while cells in an *ex vivo* SCN slice can maintain a consistent phase relationship with their neighbours over weeks and months in the absence of external cues, other tissues, or even dissociated cells of the SCN, cannot [30]. Instead, cells outside of the intact SCN, while maintaining a molecular oscillation, require regular input from external timing cues to keep this oscillation in phase with other cells in the tissue and the organism as a whole. This observation highlights the importance of entraining cues for aligning the phase of mammalian cells outside of the SCN to other cells in the same organism and to the external environment.

## Entrainment of cellular circadian rhythms

**Fig. 1:**
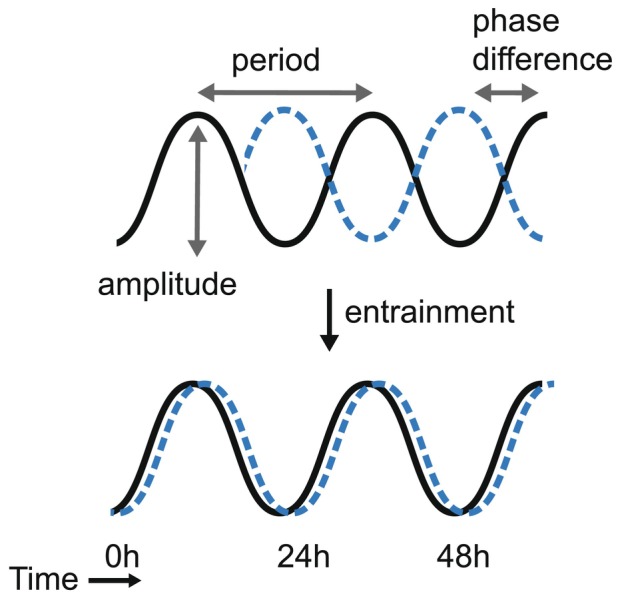
Key features of a circadian rhythm. Schematic highlighting period, amplitude, phase and phase entrainment. Black‐ and blue‐dotted lines represent two independent rhythms.

Entrainment, in a circadian context, is the alignment of a rhythmic process with an external oscillation (Fig. 1). Since the beginning of the modern circadian field, it has been clear that the major entraining cues for mammals are light, feeding and physical activity, which produce an increase in body temperature [31, 32]. For much of evolution, these three rhythmic cues have been experienced by organisms in a relatively constant phase relationship, such that they are thought to reinforce each other to promote robust alignment of organisms with the day–night cycle. Disruption of the phase relationship between these three cues, such as occurs in shift work, where workers are active and eating at night, has numerous striking health consequences, including increased rates of type‐2 diabetes and cancer [33, 34, 35]. Thus, entrainment is a critical factor not just for a well‐functioning circadian rhythm, but for overall organismal health.

When discussing mammalian cells, several different terms are used to describe entrainment at the cellular level, including phase ‘resetting’, phase ‘shifting’ and phase ‘synchronisation’ [36, 37], and these terms will be used interchangeably in this review. It should be noted, however, that in some contexts these terms may not be exactly equivalent and should therefore be clearly defined when used. This terminology reflects the fact that, unlike whole animals, cells outside of the SCN do not necessarily require exposure to multiple cycles of rhythmic cues to allow them to align the phase of their circadian oscillation but can, particularly when experiencing supraphysiological doses of entraining cues, produce up to 12‐h phase shifts in response to a single cue (Fig. 1). While such large alterations in phase are unlikely to be long‐lasting in the context of a cell within a multicellular organism, the increased capacity of non‐SCN cells to alter their phase indicates the importance of phase flexibility in many mammalian cell types.

Despite long‐standing knowledge of the three major entraining cues of light, feeding and temperature, how these cues are communicated to every individual cell in a mammal to align cellular phase remains an active area of research, the current understanding of which I will explore below. For the purposes of this review, I will only discuss these three conserved, physiological entraining cues, although readers should be aware that other, non‐physiological or more tissue‐specific cues do exist, such as treatment with the cAMP activator, forskolin [38] and mechanical stress [39, 40].

### Light

Most mammalian cells are not intrinsically photosensitive. As such, cells within a mammal rely on a small population of cells within the retina to disseminate light information to the rest of the organism. From a circadian perspective, a critical group of cells is intrinsically photosensitive retinal ganglion cells (ipRGCs), which directly detect information about light quantity and quality and communicate this to the SCN [41, 42, 43, 44]. Following assimilation of this information, which has been extensively covered in other reviews [45], the SCN then signals to the adrenal glands via the HPA‐axis [46, 47] to promote the release of steroid hormones: cortisol in humans and corticosterone in mice [48]. Responding to this glucocorticoid release is a primary mechanism by which cells outside of the SCN align their circadian phase to lighting cues.

**Fig. 2:**
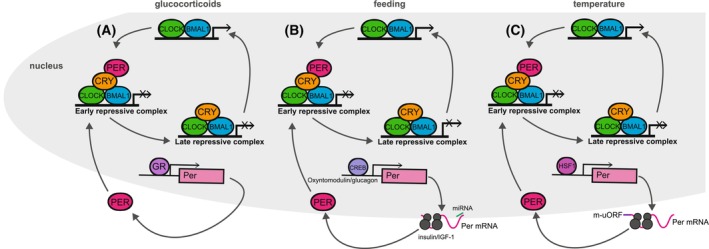
Major cellular entrainment cues acutely alter PER expression. Schematic showing the core mammalian circadian complexes and the input of the major cellular entraining cues (A) glucocorticoids, (B) feeding and (C) temperature to these TTFL complexes by altering PER expression.

Detailed cellular work has allowed for dissection of the mechanism by which glucocorticoids reset the phase of each cell individually. The most prominent mechanism is through increasing transcription of the mammalian circadian repressor protein, PERIOD (PER), of which there are three homologues, PER1, PER2 and PER3. This phenomenon was first noted in 1998 and was one of the first observations that confirmed circadian rhythms in cells in culture [16, 24]. The promoter of *Per* genes contains numerous transcriptional elements. In *Per1* and *Per2*, this has been found to include at least one glucocorticoid response element (GRE) [49, 50]. In *Per2*, this GRE overlaps with a previously annotated E‐box (the binding site for the circadian transcription factor complex, CLOCK:BMAL1), such that expression from this site is BMAL1‐dependent [51]. Accordingly, in response to glucocorticoid release, mammalian cells produce an acute induction in the expression of PER1 and PER2 [24, 51]. Notably, *Per3* has not been so clearly identified to be transcriptionally regulated by glucocorticoids, although identification of a putative GRE in the *Per3* promoter suggests that this is likely [52]. PER proteins are the limiting factor for the formation of the ‘early repressive complex’ [53, 54, 55], a complex whose formation is essential for the repressive phase of the circadian TTFL (Fig. 2). Thus, increasing PER protein abundance following increased transcription is thought to be sufficient to reset the circadian phase of cells. In line with this, at high concentrations, glucocorticoids have a strong (type‐0) phase‐resetting effect on cells in culture [37, 56]. At lower, more physiological concentrations, glucocorticoids produce a smaller, but still substantial, shift in phase, in line with their role of aligning the phase of non‐photosensitive cells with the light–dark cycle. It is important to note that the effects of glucocorticoids on phase are cell autonomous: every single cell alters its phase individually in response to the application of the cue. This is demonstrated by Manella and colleagues [56], who use both the physiologically relevant steroid corticosterone and the synthetic glucocorticoid dexamethasone to demonstrate that both achieve the same phase resetting in cells, although the concentration of dexamethasone required for a maximal response is ~ 10‐fold lower than that of corticosterone. This is commonly exploited in circadian cell biology, where dexamethasone is used to allow for a population of cells to be phase‐synchronised with their neighbours as part of many standard experimental protocols [24]. It is striking that, unlike feeding and temperature, the mechanisms by which glucocorticoids reset cellular phase have, to date, been found to be entirely transcriptionally regulated, with no post‐transcriptional or post‐translational regulation clearly identified or disproved. As techniques for studying these events advance, it would be of interest to revisit whether entrainment to glucocorticoids is purely transcriptionally driven, or if it is subject to more complex regulation.

### Food

Time‐of‐feeding is another major entraining cue in mammals. Indeed, it can be argued to be the most important entrainment cue; when food resources are scarce, habitually nocturnal mice overcome their aversion to light and become diurnal, to allow them to seek food during the day [57]. In this scenario, the expression of core circadian genes in all tissues other than the SCN shifts by up to 12 h to align with time‐of‐feeding [58]. This conflict of entraining cues highlights the importance of seeking out nutrients, even if this conflicts with entraining light cues. For 50 years after the identification of feeding as an entraining cue, it was unclear how this cue was communicated to each individual cell. Over this time, several groups had attempted to identify a specific region of the brain that detected feeding cues and communicated this timing information to other cells of the body: a so‐called ‘food‐entrainable oscillator’. Such a centralised locus of feeding detection would be analogous to the SCN; the SCN itself is not required for food entrainment [59]. While multiple candidate regions have been proposed [60], no single locus has, to date, been satisfactorily uncovered. In contrast, in recent years, a handful of key feeding cues that can entrain individual cells directly have been established unequivocally.

One set of candidates proposed to play a role in resetting cellular phase following feeding in mammals is the peptide hormones glucagon and oxyntomodulin. Like glucagon, oxyntomodulin is also derived from proglucagon and consists of glucagon and a C‐terminal octapeptide extension [61]. Critically, oxyntomodulin levels rise following feeding [62], while glucagon levels are typically most elevated following fasting [63]. Work from Landgraf *et al*. [64] showed that the application of oxyntomodulin to organotypic slices of mouse liver *ex vivo* prompted signalling, likely through the glucagon receptor (GCGR), resulting in CREB activation [65]. As has previously been described in the light response in the SCN [23, 66], this CREB activation induced expression of both PER1 and PER2 in the liver and produced an associated shift in circadian phase. A similar acute increase and phase shift in PER2 liver expression was also demonstrated following oxyntomodulin application *in vivo*. Although this work does not include investigation of cell types not found in the liver, the broad expression of the glucagon receptor [67] suggests that this mechanism may also play a role in cellular entrainment to feeding in other cell types. Glucagon itself has also been implicated in inducing PER1 and PER2 expression via CREB in mouse liver [68]. Additionally, glucagon has been proposed to stimulate BMAL1 expression in the liver, again via CREB signalling, although the effects of this on circadian phase were not explored [69].

A primary factor signalling time‐of‐feeding to cells is the feeding hormone insulin. The earliest works describing the presence of circadian rhythms in Rat1 cells in culture noted that the application of insulin drove an acute increase in PER1 and PER2 expression [25]. More recently, insulin was identified as a phase‐resetting agent in NIH3T3 cells expressing the insulin receptor ectopically, as well as in liver and white adipose tissue slices *ex vivo*, and hepatocytes in culture [70, 71]. Subsequent work extended these initial findings, demonstrating that insulin or IGF‐1 application to lung fibroblasts in culture drove an acute increase in PER1, PER2 and PER3 expression that was driven by a combination of transcriptional and post‐transcriptional mechanisms [72]. The latter of these consists of a coincidence‐detection mechanism of signalling through PI3K and mTOR, combined with the downregulation of specific PER‐regulating miRNAs [73]. This increase in PER expression drove a shift in circadian phase across all *ex vivo* tissue slices tested, ranging from kidney to testes, in line with the ubiquitous expression of the insulin receptor. Critically, inhibition of the insulin receptor in mice was sufficient to abolish behavioural food entrainment, highlighting insulin as a critical molecule communicating time‐of‐feeding in mammals through actions at the level of the single cell.

A common element between these feeding cues is that, unlike light, they do not require central co‐ordination via a locus in the brain. Instead, it appears that entrainment to time‐of‐feeding is a more disperse property, with multiple tissues—the pancreas for insulin and glucagon, the liver for IGF‐1, the gut for oxyntomodulin—producing hormonal timing cues in direct response to feeding to which other cells throughout the body respond. This series of discoveries contradicts earlier work which posited the existence of a neuroanatomical locus that controlled the mammalian capacity to entrain to time‐of‐feeding [60]. Thus, while this is still an area of active research that warrants further exploration, existing evidence would suggest that there is no ‘food‐entrainable oscillator’, and that this function is instead carried out by each cell individually in response to specific feeding hormones.

### Temperature

The third physiological cellular entraining cue is temperature. Even in homeothermic mammals, there are daily rhythms in body temperature, which are often temporally associated with feeding, as peak body temperature accompanies food intake [58]. Generally, the combination of temperature, light and feeding cycles are thought to act to reinforce each other, adding robustness to the system of circadian entrainment. In cell culture however, it is possible to disentangle these cues: early work from Steve Brown provided some key observations that cycling external temperature rhythms with an amplitude as small as 2 °C for 3–5 days is sufficient to entrain the phase of cellular circadian rhythms [36, 74], while pulses of larger temperature differentials can acutely synchronise a population of cells [75]. Work from Buhr *et al*. [76] highlighted the ubiquitous nature of temperature entrainment: as with insulin and corticosterone, all tissues other than the SCN reset their circadian phase in response to a cycle of external temperatures that fall within the physiological range.

Likely because temperature is directly detected by cells, rather than being communicated predominantly by a circulating hormonal factor like for light and feeding, current understanding suggests that there are multiple, likely combinatorial, mechanisms by which temperature changes input into circadian phase. Several groups identified heat shock factor 1 (HSF1) as a key protein regulating the cellular circadian response to both increases and decreases in temperature. Critically, there are two heat shock elements in the *Per2* promoter, mutation of which prevented the increase in PER2 expression following acute temperature shock [75]. Knock‐out of HSF1 inhibits phase synchronisation by acute temperature shock [75] and also substantially slows the ability of cells in culture to align their phase with low‐amplitude temperature cycles [76, 77]. Cold‐inducible RNA‐binding protein (CIRP) has also been shown to respond to physiological temperature cycles in cells by increasing its expression as temperatures decrease [78]. Knockdown of CIRP slowed the ability of cells to align their phase to external temperature cycles. Subsequent work has suggested that this may occur through alternative polyadenylation by CIRP and another RNA‐binding protein, RBM3, although specific circadian targets of these proteins have not been identified [79].

More recent work has identified several other, more detailed, post‐translational mechanisms by which cells shift their circadian phase in response to temperature cycles. Miyake *et al*. identified the presence of a temperature‐sensitive minimal uORF (m‐uORF) upstream of *Per2* exon 1, which caused an accumulation of ribosomes in the *Per2* 5′UTR following a 3.5 °C increase in temperature [80]. This resulted in an increase in PER2 translation following the temperature increase, while *Per2* transcript levels remained unaltered. As with corticosterone and insulin cues, this increase in PER2 is thought to increase the formation of the early repressive complex, thereby shifting the circadian phase [53, 81]. The authors subsequently show that, as for insulin, the response to temperature change is also regulated by PI3K, which may itself regulate the temperature‐sensitive *Per2* m‐uORF, although the mechanisms underlying this are not well explored. Beale *et al*. [82] propose mTOR as a further post‐translational mechanism that regulates the differential response to environmental temperature change that is exhibited by nocturnal versus diurnal mammals: cells from nocturnal animals entrain to temperature in an opposite phase to cells derived from diurnal animals. Inhibition of both mTOR and translation initiation made cells from nocturnal animals shift their phase of entrainment to match that of cells from diurnal animals, highlighting mTOR as another regulator of resultant phase following temperature entrainment. Given the apparent overlap between mechanisms of food and temperature entrainment, further research to consider if mTOR and perhaps PI3K represent general regulators of mammalian entrainment would be valuable.

## Conclusions and perspectives

It has become clear that the molecular circadian oscillation inside every single mammalian cell responds, either directly or indirectly, to the same phase‐resetting cues as whole mammals: light, feeding and temperature. Only one of these cues, light, requires central co‐ordination, in this case through the SCN. Entrainment to feeding and temperature, in contrast, is more dispersed in nature. In the case of feeding, signals are sensed and communicated by multiple organs through hormones, and in the case of temperature, directly by each cell. Overall, it is becoming increasingly apparent that whole organismal entrainment is reliant on the entrainment of each and every cell within it.

A question raised by this cell‐centred model of entrainment is whether there is substantial cell–cell communication of circadian phase information between a cell and its neighbours. This idea is implicit in numerous previous works which have posited the concept of a ‘tissue clock’, where individual organs have their own, distinct oscillator which forms a network to maintain whole‐organismal phase coherence [21, 83]. The idea of tissue clocks builds on concepts of ‘circadian organisation’, where multiple oscillatory components—in this case, tissues—within a system, all have to maintain an identical frequency and phase relationship for their proper function [28]. The existence of tissue clocks had previously been supported by the observation that SCN‐lesioned mice still show coherent rhythms in expression of the auxiliary circadian protein, REV‐ERBα in the liver *in vivo* [84]. Increasingly, however, it appears that the concept of a tissue clock may be misleading. While it is true that the phase of the circadian oscillator varies between different tissues [17, 18], there is minimal evidence that cells within any one organ are communicating their phase directly to their neighbours. The only clear example of such paracrine signalling of circadian phase outside of the SCN was uncovered in Finger *et al*. [85]. Here, the authors identified TGF‐β as being capable of communicating phase between neighbouring fibroblasts and U2OS cells in culture. This discovery answered a long‐standing question in the field to show that cell–cell communication of phase is possible, although the findings were not extended to the context of a multicellular organism. However, it seems unlikely that coupling through TGF‐β alone would be sufficient for maintaining phase coherence within an organ to produce a distinct tissue clock. Indeed, a number of earlier works that investigated cellular circadian rhythms did not identify clear phase coupling between cells in culture [26, 27, 86], although they did identify non‐rhythmic paracrine signals as being an important factor in maintaining a high amplitude circadian rhythm [87]. Instead, a more likely hypothesis is that cells primarily align their phase to entraining cues directly. Differing phases between tissues might predominantly reflect differences in the sensitivity of the cell types within that tissue to the cellular correlates of the major entraining cues of light, feeding and temperature. This hypothesis, however, remains to be rigorously tested in the context of a whole organism or heterogeneous tissue *ex vivo*. The existence, or otherwise, of ‘tissue clocks’, and how individual cellular phase is coordinated within a complex tissue is thus an exciting area for future research.

Another particularly striking implication of cellular entrainment to different phase‐resetting cues is exemplified in shift work. Here, the major entrainment cues of light, temperature and feeding occur in an unusual temporal order, as individuals are awake and active at times when they would naturally be asleep. This results in a state termed ‘circadian disruption’, which drives increased susceptibility to several diseases, including type‐2 diabetes and cancer [33, 34, 35]. While many of the detrimental effects of circadian disruption are clearly systemic, as major entraining cues are detected by each cell, it is possible that some of the consequences of circadian disruption occur at the cellular level. Indeed, existing data show that switching the temporal order of corticosterone and insulin application to mouse fibroblasts results in decreased cellular circadian amplitude [72]. Decreased amplitude is also a hallmark of whole organismal circadian disruption [72, 88, 89]. Future work to consider if this phenomenon persists across cell types, and identify the subsequent biological consequences, would significantly advance our understanding of the mechanisms underlying circadian disruption.

To conclude: Work over the last two decades has substantially advanced our understanding of how individual mammalian cells entrain their phase to the external environment. These findings are essential in enabling us to understand how mammalian biology sets the specific timing of its molecular circadian oscillator. There remains, however, a wide range of open questions. While good progress has been made identifying the cellular correlates of entraining cues, the mechanisms by which the cell translates these cues to specifically reset the phase of the cellular circadian clock are not always fully determined. Many of the currently identified entrainment mechanisms centre around the PERIOD proteins (Fig. 2), but the possibility for other core circadian proteins to input into resetting cellular phase warrants further exploration. Furthermore, how these multiple cues interact within a multicellular organism to produce their ultimate outputs in behaviour and physiological function is a complex but critical question that remains to be addressed. Whether to resolve jet lag, reduce the detrimental impacts of shift work or to better support human health during space travel [90], gaining a full understanding of cellular entrainment and its function within a whole organism will be key to safely and effectively modulating circadian rhythmicity to human benefit.

## Author contributions

PC carried out conceptualisation, visualisation, writing and funding acquisition for this work.
